# Vitamin D Deficiency and Risk of Surgical Site Infections: A Retrospective Chart Review from a Tertiary Care Center in Qatar

**DOI:** 10.3390/medsci13030163

**Published:** 2025-09-01

**Authors:** Rana Farsakoury, Ahmad Hamdan, Muhammad Naseem Khan, Habib H. Farooqui, Sara Al Harami, Susu M. Zughaier

**Affiliations:** 1 Department of Plastic and Reconstructive Surgery, Hamad Medical Corporation, Doha P.O. Box 3050, Qatar; 2 Department of Basic Medical Sciences, College of Medicine, QU Health, Qatar University, Doha P.O. Box 2713, Qatar; 3 Department of Population Medicine, College of Medicine, QU Health, Qatar University, Doha P.O. Box 2713, Qatar

**Keywords:** retrospective chart review, infectious complications, surgical site infection, 25-dihydroxyvitamin D, 25(OH)D, vitamin D

## Abstract

**Background/Objectives**: Vitamin D deficiency is common in the Middle East, where it affects about 90% of the population. 25-hydroxyvitamin D [25(OH)D]. plays a key role in immune function and antimicrobial defense. Its deficiency has been implicated in surgical site infections (SSIs) also, which lead to increased healthcare costs and morbidity. Around 60% SSIs are preventable by addressing factors like 25(OH)D levels among others. In Qatar, 55.8% of the population is 25(OH)D deficient, but no direct link has been established between 25(OH)D deficiency and SSI risk. This study aims to investigate the relationship between deficient 25(OH)D levels and SSI development in surgical patients at Hamad Medical Corporation (HMC), Qatar. **Methods**: A retrospective chart review was conducted on adult patients who underwent surgery at HMC, Qatar, between January 2021 and December 2023, with known 25(OH)D levels measured within three months before surgery. A multivariate logistic regression analysis was conducted to evaluate the relationship between 25(OH)D levels and SSIs. **Results**: This retrospective chart review included 24,097 patients, with 3818 (15.8%) being 25(OH)D deficient. The mean age of the patients was 45 years, and 55% of them were female. The proportion of SSIs was highest in the 25(OH)D deficient group (2.7%) compared to the insufficient (1.8%) and sufficient (1.9%) groups, with a *p*-value of <0.01. The mean 25(OH)D level was 23 ng/mL in the SSI group, compared to 25 ng/mL in the no SSI group, with a *p*-value of <0.01. Multivariate logistic regression analysis identified several independent risk factors for SSIs, including 25(OH)D deficiency, male gender, intermediate and major case levels, longer operative times, lower preoperative serum albumin, and contaminated and dirty wounds, all with *p*-values of <0.05. **Conclusions**: Preoperative lower 25(OH)D levels increase the risk of SSIs. This study emphasizes the importance of optimizing 25(OH)D levels before surgery to reduce the occurrence of SSIs.

## 1. Introduction

25-hydroxyvitamin D [25(OH)D] deficiency is a global health issue [1]. The Institute of Medicine categorizes 25(OH)D status into three levels. 25(OH)D levels below 12 ng/mL (30 nmol/L) indicate a deficiency, while levels between 12 and 20 ng/mL (30–50 nmol/L) are considered insufficient. Levels of 20 ng/mL (50 nmol/L) or above are regarded as sufficient [2]. The prevalence of 25(OH)D deficiency differs across regions, with some areas exhibiting higher rates of deficiency than others. Variation in the prevalence of vitamin D deficiency across regions is mainly attributable to disparities in UVB radiation exposure associated with latitude, seasonality, and geography [3]. It is particularly highly prevalent in the Middle East, where approximately 90% of the population is affected to varying degrees [4]. A recent study by Mousa et al. examined the prevalence of 25(OH)D deficiency among adults in Qatar and explored the link between 25(OH)D deficiency and various monocyte percentage to HDL cholesterol ratio. The prevalence of 25(OH)D deficiency reached 55.8%, while 25(OH)D insufficiency was observed in up to 29.9% of individuals [5,6].

Humans can obtain 25(OH)D in two forms: vitamin D3 (cholecalciferol), which is produced in the skin through Ultraviolet B radiation exposure, and vitamin D2 (ergocalciferol), which is generated in yeast and fungi upon UVB exposure. Both forms can be acquired through animal based foods, though some plants have been genetically modified to produce vitamin D3 for supplements [7]. In the liver, the enzyme CYP27A1 converts both forms into 25(OH)D, a reliable marker of vitamin D status. This 25(OH)D is then further converted into the active form, 1,25-dihydroxyvitamin D (1,25[OH]2D), mainly in the kidneys, although other tissues with the CYP27B1 enzyme can also produce it [8].

25(OH)D is a critical modulator of the immune system, exerting distinct effects on innate and adaptive immunity. The importance of 25(OH)D and its active form, 1,25(OH)_2_D_3_, in immune function became clear with the discovery of the 25(OH)D receptor (VDR) in activated inflammatory and immune cells. 1,25(OH)_2_D_3_ enhances innate immune defenses by inducing antimicrobial peptides, including cathelicidin (CAMP/LL-37) and defensins, which disrupt microbial membranes and facilitate pathogen clearance. In addition, 1,25(OH)_2_D_3_ promotes antibacterial mechanisms such as reactive oxygen species generation and autophagy [9]. Within adaptive immunity, 1,25(OH)_2_D_3_ directly influences T-cell activity by affecting T-cell proliferation, cytokine production, and the development of Th1, Th17, Th2, and regulatory T-cells. A key aspect of immune regulation involves its effects on myeloid dendritic cells (DCs), where it downregulates costimulatory molecules (CD40, CD80, CD86) and IL-12, while boosting IL-10 levels. This promotes the creation of tolerogenic DCs, reducing Th1 cell development, enhancing CD4+ suppressor T-cell activity, and facilitating regulatory T-cell recruitment via increased CCL22 expression. Plasmacytoid DCs are less influenced by VDR agonists, which means their tolerogenic potential remains largely unaffected. These immune-modulating actions of 1,25(OH)2D have prompted interest in its potential use in treating autoimmune diseases. Furthermore, 1,25-dihydroxyvitamin D3 inhibits B-cell proliferation, immunoglobulin production, and the differentiation of B-cell precursors into plasma cells [10]. Macrophages, DCs, and T-cells also regulate the synthesis and breakdown of 1,25(OH)2D. Collectively, these mechanisms position 25(OH)D as a key regulator of host defense, augmenting antimicrobial capacity while maintaining immune homeostasis [8]. Vitamin D functions as an immune regulator with context-dependent effects that can be either beneficial or potentially problematic. Its capacity to modulate adaptive immune responses, particularly by promoting regulatory mechanisms and reducing excessive inflammatory responses, may help prevent or alleviate autoimmune diseases such as multiple sclerosis and rheumatoid arthritis, though causality is still being established through clinical research. Simultaneously, vitamin D enhances certain aspects of innate immunity by stimulating the production of antimicrobial peptides like cathelicidin, thereby providing protection against pathogens [11]. However, its immunomodulatory actions could, under certain circumstances, potentially impair optimal adaptive immune responses against infections [9,12], highlighting the importance of balanced vitamin D status.

Surgical site infections (SSIs) are wound infections that occur within 30 days of surgery or up to a year post-surgery if an implant is inserted [13]. SSIs are among the most common healthcare-associated infections (HAIs), affecting 2.5% of surgical procedures globally [14]. A recent study at Hamad Medical Corporation (HMC) in Qatar evaluated the incidence of surgical site infections (SSIs) in patients who underwent appendectomy, herniorrhaphy, and caesarean section procedures. Based on an analysis of 5127 surgeries performed between 2013 and 2023, the study reported SSI rates of 2.19% for appendectomy, 2.53% for herniorrhaphy, and 2.56% for caesarean sections [15].These infections impose a significant burden on patients and healthcare systems, contributing to the spread of antimicrobial resistance (AMR), increased morbidity and mortality rates, prolonged hospital stays, and higher healthcare costs [16]. However, up to 60% of SSIs are preventable by addressing modifiable perioperative factors, including 25(OH)D levels [17]. Youssef et al. highlighted the potential role of 25(OH)D in reducing the risk of developing various types of hospital-acquired infections (HAIs) [18]. A systematic review and meta-analysis found higher odds of SSI with 25(OH)D levels below 20 ng/mL (OR 1.42, 95% CI: 0.80–2.05; I^2^ = 37.2%, *p* = 0.18) and below 30 ng/mL (OR 3.84, 95% CI: 2.13–5.56; I^2^ = 0.0%, *p* = 0.83) [19].

In Qatar, 25(OH)D deficiency is highly prevalent, affecting approximately 71.4% of the population [5]. Currently, there is lack of evidence determining the relationship between 25(OH)D levels and SSI risk in Qatar. This study aims to examine whether preoperative 25(OH)D deficiency can lead to an increased risk of SSIs among surgical patients at Hamad Medical Corporation (HMC) in Qatar. Establishing this relationship could inform perioperative protocols and reduce postoperative complications in a potentially high-risk population.

## 2. Materials and Methods

### 2.1. Study Design and Settings

This study was a retrospective chart review and was conducted in accordance to the Strengthening the Reporting of Observational Studies in Epidemiology (STROBE) guidelines [20]. Ethical approval was obtained from the Ethics Committee of HMC (MRC-01-23-662) and Qatar University (IRBNeT ID: 2133381-1).

The study was conducted at the in-patient, out-patient, and emergency setting at the Department of Surgery, Hamad Medical Corporation—a tertiary referral center located in Qatar. The nature of surgical cases varied between emergent and elective. The research collected data over a three-year period, from 2021 to 2023.

### 2.2. Patients’ Selection

This study was conducted on patients who underwent any type of surgery at HMC, Qatar, between 1 January 2021 and 31 December 2023. Data were retrospectively extracted from the electronic health records (EHR) system (Cerner) at HMC. The collected information included patient demographics such as age, gender, nationality, height, weight, body mass index (BMI), and documented chronic medical conditions. Admission-related data encompassed the dates of hospital presentation, surgery, and discharge; the type, site, and duration of the surgical procedure; use of antibiotic prophylaxis; medications administered; and the overall duration of hospitalization. Preoperative laboratory investigations included complete blood count (CBC), comprehensive metabolic panel (CMP), blood glucose, procalcitonin, lipid profile, and 25(OH)D levels. Additionally, data regarding the presence or absence of surgical site infections (SSI) were collected, along with corresponding microbiology laboratory results when available.

Study participants were included based on the following criteria: (1) age over 18 years, (2) underwent surgery at HMC, Qatar during the specified period, and (3) had a documented 25(OH)D levels measured within three months prior to surgery reflecting the half-life of 25(OH)D. Exclusion criteria included: (1) immunocompromised status and (2) established diagnosis of malignancy.

### 2.3. Study Outcomes

The primary outcome of our study was to determine the incidence of SSI between 25(OH)D categories. The diagnosis of surgical site infections (SSIs) was confirmed when it met the standardized definition established by the National Clinical Guidelines in Qatar. The infection should be detected within 30 days of surgery and up to one year of surgery if an implant is inserted. When any signs or symptoms of infection are present, further diagnostic investigations are warranted. This includes collecting wound swabs, synovial fluid, or tissue samples for microbiological culture, keeping in mind that negative results do not definitively rule out infection. Based on clinical suspicion and laboratory investigations, a diagnosis of SSI was documented on the electronic medical record which was extracted to determine the presence of SSI in the dataset [21].

The secondary objective was to identify and assess a predefined set of risk factors as potential preoperative predictors of SSI. Risk factors of interest (Age, BMI, gender, diabetes, hypertension, case level, operative time, preoperative serum albumin and wound classification) were selected based on a comprehensive literature review.

25(OH)D levels were assessed using samples collected within three months of the surgical procedure to accurately represent the patient’s perioperative 25(OH)D status. This timeframe was selected based on the pharmacokinetics of 25(OH)D, which is the principal circulating form of vitamin D, that possesses a half-life of approximately 2 to 3 weeks and remains relatively stable in circulation for up to three months [22]. Quantification of serum 25(OH)D was performed using the enzyme-linked immunosorbent assay (ELISA) method, a validated and commonly used technique. All analyses were conducted at Hamad Medical Corporation. This standardized methodology supported the reliability and comparability of 25(OH)D measurements across all participants.

Serum 25(OH)D levels were categorized according to the institute of Medicine as follows: <12 ng/mL in 3818 patients (15.8%), 12–20 ng/mL in 6736 patients (28.0%), and >30 ng/mL in 13,543 patients (56.2%) [2].

The model was developed based on a predefined set of risk factors identified a priori, which included preoperative albumin, hypertension, age, dyslipidemia, gender, length of hospital stay (LOS), diabetes, body mass index (BMI), case complexity, duration of surgery, and wound classification.

### 2.4. Statistical Analysis

Statistical analysis was performed using STATA version 18 (College Station, TX, USA) [23]. Reported demographic and clinical data included: age (in years), gender, nationality, BMI (in kg/m^2^), smoking status, ASA classification and preoperative labs. Categorical variables were presented as numbers and percentages, while continuous variables were expressed as means and standard deviations. Differences between categorical variables were assessed using Pearson’s chi-square test, while differences in continuous variables were evaluated using Student’s *t*-tests or ANOVA, as appropriate.

Using the Daggity tool (version 3.1) [24], a Directed Acyclic Graph (DAG) was created to identify a minimal set of covariates for the regression model (Figure S1). These confounding factors were age, BMI, gender, diabetes, hypertension, dyslipidemia, case level, operative time, preoperative albumin, length of hospital stay (LOS), and wound classification. Multivariable logistic regression analysis was conducted to examine the association between 25(OH)D levels, SSI risk, and predictors. Results were reported as odds ratios (OR) with corresponding 95% confidence intervals (CI) and *p*-values. *p*-values were used to assess the degree of evidence against the model hypothesis.

## 3. Results

The study population comprised 24,097 patients who underwent various types of surgery, with 13,719 (55%) being female. Fifty five percent of the 25(OH)D deficiency group were women. The mean age of the patients was 45 years. Patient characteristics based on 25(OH)D status are summarized in Table 1.

**Figure 1:**
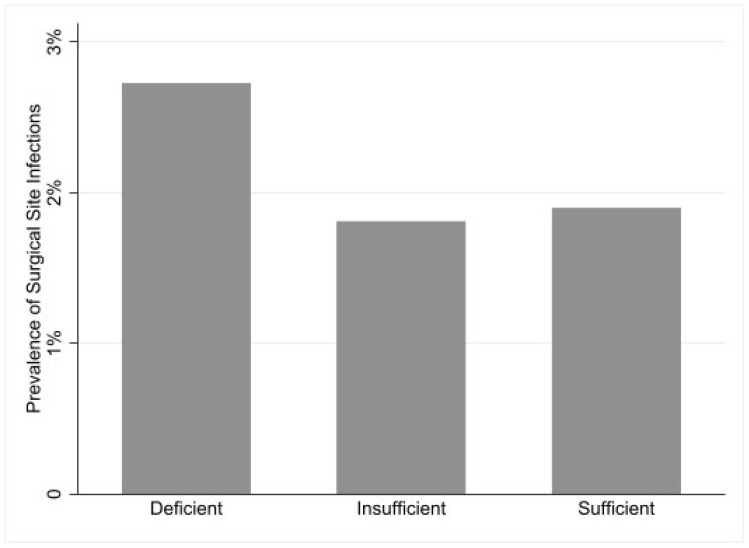
Incidence of surgical site infection based on 25(OH)D Status.

**Figure 2:**
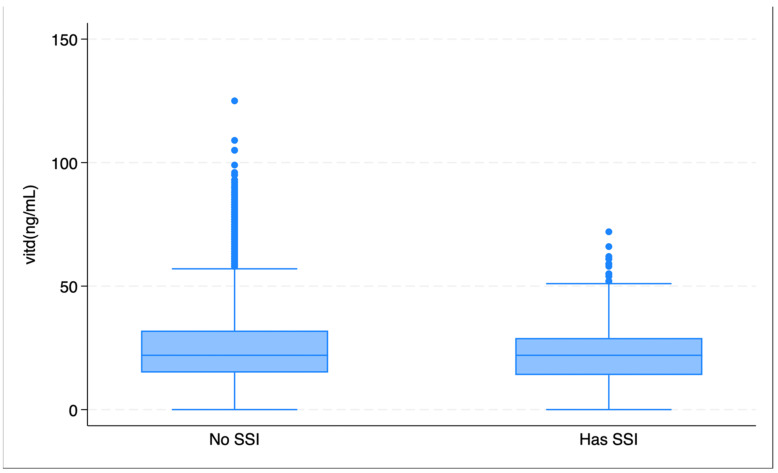
Boxplot showing the variation in 25(OH)D levels within SSI categories.

Overall, surgical site infections (SSIs) were observed in 483 patients (2%). The mean serum 25(OH)D level was 23 ng/mL in the SSI group compared to 25 ng/mL in the non-SSI group. Patient characteristics based on SSI status and surgical factors are detailed in Table 2. The incidence of SSI was highest in the 25(OH)D deficient group (2.7%), compared to 1.8% in the insufficient group and 1.9% in the sufficient group (*p* < 0.01; Figure 1). A boxplot displays 25(OH)D levels in patients with and without SSI (Figure 2).

Adjusted multivariate logistic regression analysis was conducted to evaluate the association between serum 25(OH)D level and occurrence of SSI (Table 3). To account for potential confounding effects, the model was controlled for age, gender, body mass index (BMI), diabetes status, hypertension, case complexity, duration of surgery, preoperative albumin levels, and wound classification, all identified using DAG (Figure S1). 25(OH)D deficiency was associated with increased odds of SSI after surgery (odds ratio [OR], 1.39; 95% confidence interval [CI], 1.04–1.86; *p* = 0.02). The association between variables of interest and SSI incidence was evaluated in a predictive model (Table 4). Male patients had higher odds of SSI compared to female patients (OR, 1.47; 95% CI, 1.15–1.88; *p* < 0.05). Longer operative times were linked to increased SSI risk (OR, 1.21; 95% CI, 1.12–1.31; *p* < 0.05). Contaminated and dirty wounds had higher odds of SSI compared to clean and clean-contaminated wounds (OR, 3.42; 95% CI, 2.14–5.45; *p* < 0.05, and OR, 1.99; 95% CI, 1.34–2.97; *p* < 0.05, respectively). High preoperative serum albumin was associated with lower odds of SSI (OR, 0.91; 95% CI, 0.89–0.92; *p* < 0.05). Age, BMI, diabetes, hypertension, and case level were not identified as independent risk factors for SSIs.

## 4. Discussion

25(OH)D deficiency is a known risk factor for postoperative complications, especially wound infections following surgery [25]. Our study demonstrated that 25(OH)D deficiency was associated with increased risk of SSIs in Qatar. This relationship appeared stronger in men than in women, as well as in intermediate and major case levels, longer operative times, lower preoperative serum albumin, and contaminated and dirty wounds. Additionally, no significant association was observed between age, BMI, diabetes, and hypertension in relation to SSIs.

This finding aligns with eight other studies that reported a similar association [26,27,28,29,30,31,32,33]. This relationship may be explained by the immunomodulatory effects of 25(OH)D. 25(OH)D enhances the innate immune system by inducing the production of antimicrobial peptides, particularly LL-37. The active form of 25(OH)D, 1,25-dihydroxyvitamin D3, activates the vitamin D receptor (VDR), which in turn stimulates the CAMP gene responsible for LL-37 production. LL-37 not only kills pathogens by disrupting their membranes but also modulates immune responses by regulating cytokine production and attracting immune cells. These functions underscore 25(OH)D’s critical role in both antimicrobial defense and immune system regulation [14]. 25(OH)D plays a role in the immune system by promoting chemotaxis, autophagy, and phagolysosomal function in innate immune cells, while also increasing the presence of 25(OH)D receptors on B and T lymphocytes and macrophages [34]. 25(OH)D impacts immune function, endothelial and mucosal activity, as well as glucose and calcium metabolism [35]. Additionally, other studies have shown that 25(OH)D directly affects immune cells, with its deficiency linked to higher rates of infectious diseases and the onset or progression of autoimmune disorders [11]. Consistent with our findings, both Leong and Cheng et al. reported that prolonged operative time is associated with an increased risk of surgical site infections (SSI) [36,37]. Similarly, studies by Ortega, Ferraz, Eisenberg, and Onyekwelu et al. demonstrated a strong association between wound classification and SSI risk, with contaminated and dirty-infected wounds linked to postoperative infection rates of 10–17% and over 27%, respectively] [38,39,40,41]. In addition, a systematic review and meta-analysis by Zhang et al. identified low serum pre-albumin levels as a significant and independent predictor of SSI risk [42].

In contrast to these results, some studies in the literature failed to show an association between 25(OH)D and SSIs [43,44], which can be attributed to several factors. Most of these studies were observational, and 25(OH)D levels were not consistently measured, resulting in small sample sizes and a lack of statistical power. Additionally, one of these studies focused only on one type of SSI (superficial SSIs) while examining the association [43].

Our study has several strengths. It is the first retrospective cohort study to focus on the association between 25(OH)D levels and the risk of developing SSIs in surgical patients across all specialties. It also includes a large cohort sample size and adheres to the recommendations of the STROBE guidelines [20]. However, it has limitations, as it is an observational study and is therefore susceptible to selection bias. Another limitation pertains to potential unmeasured confounders, such as physical activity levels and dietary habits, which could not be accounted for due to the unavailability of these data in chart reviews. Still, further well-designed randomized controlled trials (RCTs) are warranted to rigorously evaluate the impact of preoperative assessment and optimization of 25(OH)D status on the risk of developing SSIs under more controlled conditions. Additionally, more retrospective cohort studies are necessary to assess the impact of 25(OH)D on different clinical and economic outcomes in surgical patients.

## 5. Conclusions

An association was found between low 25(OH)D levels in surgical patients and an increased risk of developing surgical site infections (SSIs) across various types of surgeries. As SSIs form a great burden for patients and the health care system [16], it is worth checking and optimizing 25(OH)D levels preoperatively to decrease its burden.

## Figures and Tables

**Table 1:** Characteristics of patients based on 25(OH)D status.

Variable		Total Number Patients N = 24,097			*p*-Value
		Deficient	Insufficient	Sufficient	
N (%)		3818 (15.8%)	6736 (28.0%)	13,543 (56.2%)	
Gender, *n* (%)	Female	2098 (55.0%)	3624 (53.8%)	7997 (59.0%)	<0.01
	Male	1720 (45.0%)	3112 (46.2%)	5546 (41.0%)	
Age(years), mean ± SD		38 ± 13	42 ± 14	49 ± 15	<0.01
Nationality	Non-Qatari	2345 (61.4%)	4547 (67.5%)	8143 (60.1%)	<0.01
	Qatari	1473 (38.6%)	2189 (32.5%)	5400 (39.9%)	
Smoking status, *n* (%)	No	3100 (81.2%)	5465 (81.1%)	11,427 (84.4%)	<0.01
	Yes	718 (18.8%)	1271 (18.9%)	2116 (15.6%)	
BMI, mean ± SD		31 ± 8	30 ± 7	30 ± 7	<0.01
Comorbidities					
Diabetes, *n* (%)	No	3631 (95.1%)	6422 (95.3%)	12,589 (93.0%)	<0.01
	Yes	187 (4.9%)	314 (4.7%)	954 (7.0%)	
Hypertension, *n* (%)	No	3692 (96.7%)	6433 (95.5%)	12,699 (93.8%)	<0.01
	Yes	126 (3.3%)	303 (4.5%)	844 (6.2%)	
Dyslipidemia, *n* (%)	No	3754 (98.3%)	6552 (97.3%)	12,860 (95.0%)	<0.01
	Yes	64 (1.7%)	184 (2.7%)	683 (5.0%)	
ASA Classification, *n* (%)		0 (0.0%)	2 (<1%)	6 (<1%)	<0.01
	1	872 (22.8%)	1617 (24.0%)	3352 (24.8%)	
	2	2178 (57.0%)	3936 (58.4%)	7323 (54.1%)	
	3	686 (18.0%)	1043 (15.5%)	2613 (19.3%)	
	4	80 (2.1%)	138 (2.0%)	237 (1.7%)	
	5	2 (0.1%)	0 (0.0%)	12 (0.1%)	
Preoperative Labs					
WBC, mean ± SD		10 ± 4	10 ± 3	10 ± 4	<0.01
Neutrophils, mean ± SD		7.3 ± 3.5	7.2 ± 3.4	6.9 ± 3.3	<0.01
Hemoglobin, mean ± SD		11.4 ± 2.1	11.4 ± 2.0	11.4 ± 1.9	0.46
CRP, mean ± SD		16 ± 39	7.6 ± 26	11 ± 32	0.21
ALT, mean ± SD		33 ± 48	35 ± 107	34 ± 94	0.86
AST, mean ± SD		33 ± 134	32 ± 146	32 ± 135	0.99
Albumin, mean ± SD		32 ± 7	32 ± 6	31 ± 6	<0.01
Creatinine, mean ± SD		91 ± 118	95 ± 124	113 ± 157	<0.01
Calcium, mean ± SD		2.21 ± 0.15	2.22 ± 0.14	2.21 ± 0.15	0.13

**Table 2:** Characteristics of patients based on surgical site infection status and surgical factors.

Variable		SSI		*p*-Value
		Negative	Positive	
N (%)		23,614 (98%)	483 (2%)	
25(OH)D (ng/mL), mean ± SD		25 ± 13	23 ± 13	<0.01
Admission type	Elective	18,280 (77.4%)	202 (41.8%)	<0.01
	Emergency	5334 (22.6%)	281 (58.2%)	
Wound class	Clean	12,534 (53.1%)	212 (43.9%)	<0.01
	Clean-Contaminated	9805 (41.5%)	174 (36.0%)	
	Contaminated	506 (2.1%)	39 (8.1%)	
	Dirty-Infected	769 (3.3%)	58 (12.0%)	
LOS (days), mean ± SD		2.8 ± 6.4	25 ± 34	<0.01
ICU	No	22,363 (94.7%)	314 (65.0%)	<0.01
	Yes	1251 (5.3%)	169 (35.0%)	
ICU LOS (days), mean ± SD		4.2 ± 5.9	16.6 ± 20.5	<0.01
Case level	Minor	7351 (31.1%)	178 (36.9%)	0.02
	Intermediate	2491 (10.5%)	53 (11.0%)	
	Major	13,772 (58.3%)	252 (52.2%)	
Preoperative antibiotics	No	21,644 (91.7%)	382 (79.1%)	<0.01
	Yes	1970 (8.3%)	101 (20.9%)	
Operative time (hours), mean ± SD		1.2 ± 3.0	1.7 ± 1.6	<0.01
Type of surgery	Cardiothoracic Surgery	265 (1.1%)	21 (4.3%)	<0.01
	ENT	1090 (4.6%)	8 (1.7%)	
	General Surgery	6506 (27.6%)	212 (43.9%)	
	Gynecology	1524 (6.5%)	25 (5.2%)	
	Neurosurgery	391 (1.7%)	41 (8.5%)	
	Obstetrics	2481 (10.5%)	13 (2.7%)	
	Ophthalmology	2514 (10.6%)	1 (0.2%)	
	Oral and Maxillofacial Surgery	221 (0.9%)	5 (1.0%)	
	Orthopedic Surgery	2183 (9.2%)	60 (12.4%)	
	Plastic and Reconstructive Surgery	2035 (8.6%)	67 (13.9%)	
	Urology	3213 (13.6%)	11 (2.3%)	
	Vascular Surgery	1191 (5.0%)	19 (3.9%)	

**Table 3:** Adjusted Multivariate Logistic regression analysis to determine risk factors related to Surgical Site Infections.

Surgical Site Infections	Adjusted Odds Ratio *	95% Confidence Interval	*p*-Value
25(OH)D Status:			
Sufficient	Reference value	-	-
Insufficient	1.09	0.83–1.43	0.52
Deficient	1.39	1.04–1.86	0.02

* Adjusted for Age, BMI, gender, diabetes, hypertension, case level, operative time (hours), preoperative serum albumin (gm/L) and wound class. Minimal set of variables for adjustment was determined using DAG.

**Table 4:** Multivariate logistic regression for the association between variables of interest and SSI.

Surgical Site Infections	Odds Ratio	95% Confidence Interval	*p*-Value
25(OH)D Status:			
Sufficient	Reference value	-	-
Insufficient	1.09	0.83–1.43	0.52
Deficient	1.39	1.04–1.86	0.02
Age:			
	1.01	1.00–1.02	0.03
BMI:			
	0.99	0.98–1.01	0.82
Gender:			
Female	Reference value	-	-
Male	1.47	1.15–1.88	<0.05
Diabetes:			
No Diabetes	Reference value	-	-
Has Diabetes	1.34	0.81–2.20	0.24
Hypertension:			
No Hypertension	Reference value	-	-
Has Hypertension	1.31	0.78–2.20	0.30
Case Level:			
Minor	Reference value	-	-
Intermediate	0.65	0.43–0.99	0.04
Major	0.58	0.43–0.77	<0.05
Operative Time (Hours):			
	1.21	1.12–1.31	<0.05
Pre-Operation Serum Albumin (gm/L):			
	0.91	0.89–0.92	<0.05
Wound Class:			
Clean	Reference value	-	-
Clean-Contaminated	0.95	0.73–1.25	0.75
Contaminated	3.42	2.14–5.45	<0.05
Dirty-Infected	1.99	1.34–2.97	<0.05

## Data Availability

The data for this study are under agreement with Hamad Medical Corporation and cannot be shared with third party.

## Supplementary Materials

The following supporting information can be downloaded at: https://www.mdpi.com/article/10.3390/medsci13030163/s1, Figure S1: Directed Acyclic Graph (DAG) was utilized to identify variables controlled for during the design and analysis phases.

## Abbreviations

The following abbreviations are used in this manuscript:

SSI	Surgical Site Infection
HAIs	Hospital-acquired infections
HMC	Hamad Medical Corporation
