# Supplementary material for: Vitamin D Deficiency and Risk of Surgical Site Infections: A Retrospective Chart Review from a Tertiary Care Center in Qatar
Source: Med Sci (Basel). 2025 Sep 1;13(3):163. doi: 10.3390/medsci13030163 (PMC12452610; doi:10.3390/medsci13030163)
Supplement: Supplementary file 1 [file medsci-13-00163-s001.zip › medsci-3836185-supplementary.pdf]

Vitamin D Deficiency and Risk of Surgical Site Infections: A Retrospective Chart Review from a Tertiary Care Center in Qatar

Rana Farsakoury [1,2], Ahmad Hamdan [2], Muhammad Naseem Khan [3], Habib H. Farooqui [3], Sara Al Harami [1] and Susu M. Zughaier [2,*]

[1] Department of Plastic and Reconstructive Surgery, Hamad Medical Corporation, Doha P.O. Box 3050, Qatar; rfarsakoury@hamad.qa (R.F.); salharami@hamad.qa (S.A.H.)
[2] Department of Basic Medical Sciences, College of Medicine, QU Health, Qatar University, Doha P.O. Box 2713, Qatar; ah1904442@qu.edu.qa
[3] Department of Population Medicine, College of Medicine, QU Health, Qatar University, Doha P.O. Box 2713, Qatar; naseem@qu.edu.qa (M.N.K.); hfarooqui@qu.edu.qa (H.H.F.)
* Correspondence: szughaier@qu.edu.qa

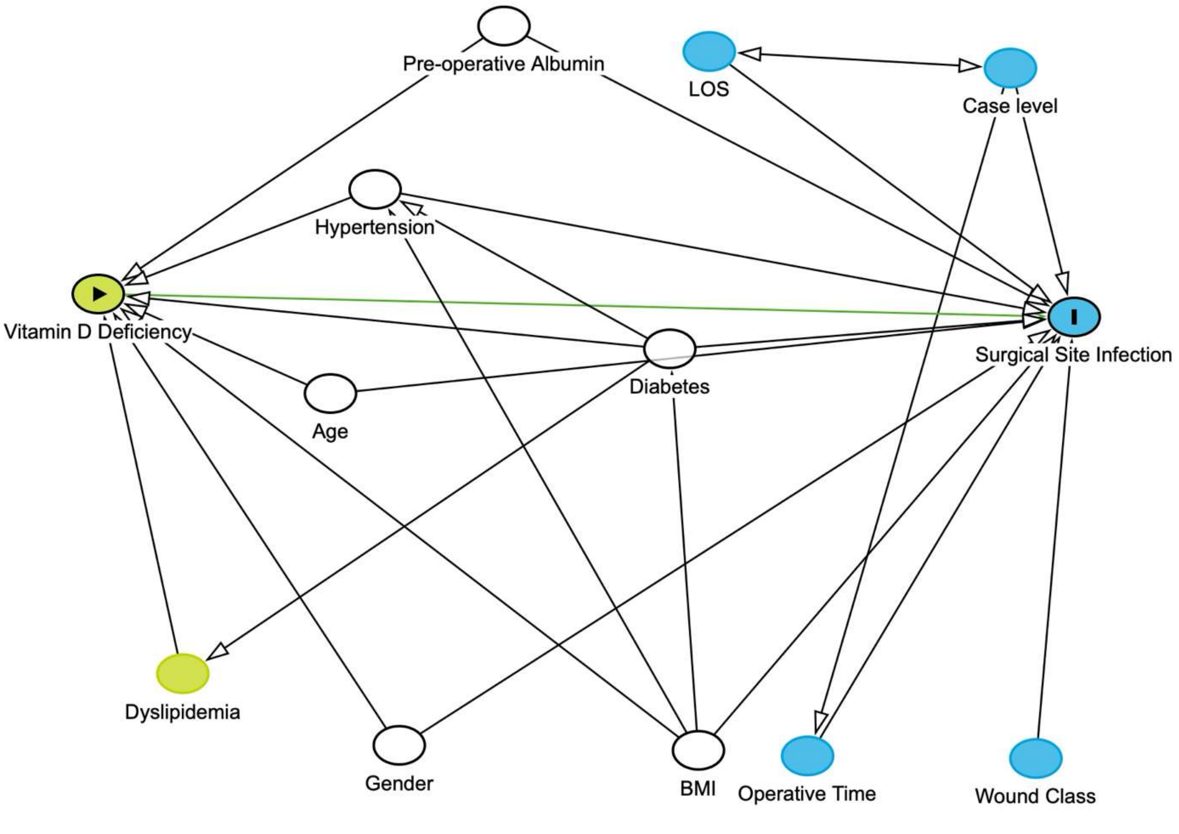

Figure S1. Directed Acyclic Graph (DAG) was utilized to identify variables controlled for during the design and analysis phases.
